# Excess Single-Stranded DNA Inhibits Meiotic Double-Strand Break Repair

**DOI:** 10.1371/journal.pgen.0030223

**Published:** 2007-11-30

**Authors:** Rebecca Johnson, Valérie Borde, Matthew J Neale, Anna Bishop-Bailey, Matthew North, Sheila Harris, Alain Nicolas, Alastair S. H Goldman

**Affiliations:** 1 Department of Molecular Biology and Biotechnology, University of Sheffield, Sheffield, United Kingdom; 2 Institut Curie, Centre de Recherche, Recombinaison et Instabilite Genetique UMR7147 CNRS Université P. et M. Curie, Paris, France; National Cancer Institute, United States of America

## Abstract

During meiosis, self-inflicted DNA double-strand breaks (DSBs) are created by the protein Spo11 and repaired by homologous recombination leading to gene conversions and crossovers. Crossover formation is vital for the segregation of homologous chromosomes during the first meiotic division and requires the RecA orthologue, Dmc1.We analyzed repair during meiosis of site-specific DSBs created by another nuclease, *VMA1*-derived endonuclease (VDE), in cells lacking Dmc1 strand-exchange protein. Turnover and resection of the VDE-DSBs was assessed in two different reporter cassettes that can repair using flanking direct repeat sequences, thereby obviating the need for a Dmc1-dependent DNA strand invasion step. Access of the single-strand binding complex replication protein A, which is normally used in all modes of DSB repair, was checked in chromatin immunoprecipitation experiments, using antibody against Rfa1. Repair of the VDE-DSBs was severely inhibited in *dmc1*Δ cells, a defect that was associated with a reduction in the long tract resection required to initiate single-strand annealing between the flanking repeat sequences. Mutants that either reduce Spo11-DSB formation or abolish resection at Spo11-DSBs rescued the repair block. We also found that a replication protein A component, Rfa1, does not accumulate to expected levels at unrepaired single-stranded DNA (ssDNA) in *dmc1*Δ cells. The requirement of Dmc1 for VDE-DSB repair using flanking repeats appears to be caused by the accumulation of large quantities of ssDNA that accumulate at Spo11-DSBs when Dmc1 is absent. We propose that these resected DSBs sequester both resection machinery and ssDNA binding proteins, which in wild-type cells would normally be recycled as Spo11-DSBs repair. The implication is that repair proteins are in limited supply, and this could reflect an underlying mechanism for regulating DSB repair in wild-type cells, providing protection from potentially harmful effects of overabundant repair proteins.

## Author Summary

During meiosis, DNA is deliberately damaged by formation of double-strand breaks. Programmed breaks must be repaired for cell division to be completed. Break repair enables reciprocal exchange between parental chromosomes, and this exchange acts as a link between chromosomes before anaphase separation. These links are essential to ensure that maternal and paternal chromosomes segregate into different daughter cells. Meiosis has special mechanisms to ensure the repair creates sufficient reciprocal exchanges between parental chromosomes; Dmc1 protein is essential for these mechanisms to work. When Dmc1 is absent, programmed breaks accumulate with excess single-stranded DNA nearby. Using reporter constructs integrated into yeast, we examined repair of an experimentally induced break expected not to need Dmc1. When Dmc1 is absent, programmed breaks accumulate in single-stranded form, and the experimental break is not repaired. Either preventing formation of programmed breaks, or stopping DNA near them from becoming single-stranded, relieves this repair block. We conclude that repair proteins are likely to be in limited supply during meiosis, and they run out in cells lacking Dmc1 function. Limiting protein supply may be an important regulatory mechanism, protecting DNA from potentially damaging effects of oversupply.

## Introduction

In most organisms the success of meiosis is dependent on the creation of molecular joints that serve to lock homologous chromosomes together until they mediate ordered chromosome segregation at the first meiotic division. This is achieved by the creation of crossovers, which creates a covalent link between nonsister chromatids, and through the forces of sister chromatid cohesion to maintain a link between chromosome pairs until first anaphase (reviewed in [1]). Crossovers are formed during repair of programmed DNA double-strand breaks (DSBs) created by the Spo11 protein [2,3]. DSBs can also be repaired by use of the sister chromatid as template. But, because intersister repair does not create links between homologous chromosomes, meiotic cells have evolved a strong bias toward using the homologous chromosome as donor template. Much attention is currently focused on understanding how the meiotic cell enforces the preference for interhomolog repair.

Various proteins have been implicated in directing DSB repair toward the homologous chromosome and/or away from the sister chromatid. These include the meiosis-specific RecA homolog Dmc1. In the absence of Dmc1, DNA joint molecules between homologous chromosomes fail to form, causing unrepaired DSBs to accumulate [4,5]. Also implicated in enforcing interhomolog DNA repair are members of a meiosis-specific complex, Mek1-Hop1-Red1. Mek1 is a kinase with similarities to Rad53. Loss of Mek1 function bypasses the requirement for Dmc1, rendering meiotic DSB repair Rad54-dependent and increasing the frequency of intersister recombination events [6–9]. Hop1 and Red1 are phosphor-proteins that localize to meiotic chromosome axes, and mutants of both genes have been recovered in screens for increased intersister chromatid repair [6,8,10–13].

In addition to the classical homologous recombination mechanisms, DSBs can be repaired by a second homology-dependent mechanism, single-strand annealing (SSA) [14,15]. DSBs that form during mitotic or meiotic growth are resected to create short tracts of 3′ ending single-stranded DNA (ssDNA). If unrepaired by strand invasion, resection can extend for many kilobases. Extensive resection has the potential to uncover repeated sequences that flank the initial lesion, such that complementary strands anneal leaving a flap of intervening DNA that is removed by Rad1/Rad10 flap endonuclease activity [16]. Little is known about proteins that initiate resection or catalyze the formation of long tracts of ssDNA. The Mre11 complex and Exo1 certainly contribute to resection, and these proteins influence the likelihood of DSBs repairing through SSA [17–22] (R. Johnson, M. J. Neale, A. S. H. Goldman, unpublished data). Mitotic studies show SSA to be independent of *RAD51*, *RAD54*, *RAD55*, and *RAD57*, but dependent on *RAD52* [17,18]. The single-stranded binding protein complex replication protein A (RPA) is also required for SSA, probably to help recruit Rad52 [19,23].

The activities of general and meiosis-specific recombination proteins are not restricted to repair of Spo11-induced DSBs during meiosis. This has been determined from studies of recombination induced by the HO-endonuclease and the meiosis-specific homing *VMA1*-derived endonuclease, VDE. Unlike Spo11, HO-endonuclease and VDE have strict cleavage sequence-specificity and do not become covalently bound to the DSB end, creating “clean” DSBs [2,24–27]. Despite these differences, the genetic requirements for repair of HO- and VDE-induced DSBs are similar to those of Spo11-DSBs [28–30]. For example, *SAE2* is required for removal of covalently bound Spo11 from DSB ends and for single-strand resection, and VDE-DSB repair is slowed in *sae2* mutants because resection at VDE-DSBs is also retarded [28,31] (A. Bishop-Bailey, A. S. H. Goldman, unpublished data). That Sae2 would be important for repair of a clean DSB is supported by more recent studies on HO-DSBs in mitotic cells [32]. *DMC1* is required for gene conversion at a VDE-DSB, indicating that the commitment of meiotic cells to repair using a homologous chromosome template is not restricted to Spo11-induced DSBs [28]. Studying DSBs created by an endonuclease other than Spo11 provides insight into the regulatory significance of large numbers of Spo11-DSBs and allows study of mutations at a stage beyond the point where a phenotypic block would be observed at Spo11-DSBs [31].

Here we report that the requirement of Dmc1 for meiotic DSB repair persists even when the VDE-DSB is flanked by direct repeats, which allow repair without need for DNA strand invasion. If repair by interhomolog gene conversion is precluded, and SSA is the only pathway for VDE-DSB repair, DSB repair is still strongly Dmc1-dependent. However, SSA-mediated VDE-DSB repair becomes Dmc1-independent in the absence of active Spo11, Hop1, or Sae2, all of which influence levels of resected Spo11-DSBs. Titrating DSB-associated ssDNA using hypomorphic *spo11* alleles increases SSA repair efficiency in the *dmc1*Δ cells. Analysis of Rfa1 binding supports the view that extensive Spo11-DSB-associated ssDNA formed in the absence of Dmc1 reduces the availability of ssDNA binding proteins necessary for SSA. The implication is that availability of proteins to perform DSB repair is limited, such that mutations or physiological conditions that alter the genomic distribution and/or timing of DSB formation may have unforseen pleiotropic consequences.

## Results

**Figure 1:**
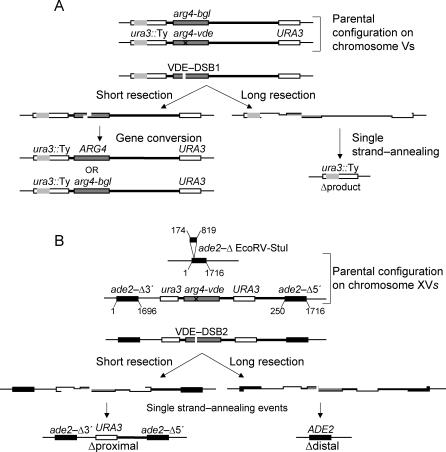
The *arg4*-vde-Containing Reporter Constructs (A) The *ura3::arg4*-vde reporter cassette containing the VDE-DSB1 site has been described previously [31]. This cassette is in a heterozygous state with a nearly identical insertion containing the *arg4*-bgl allele [69] on the opposite Chromosome V. Repair of the VDE-DSB1 is possible by gene conversion after short resection. Long resection of approximately 2 kb and 6.5 kb on the left and right of the VDE-DSB uncover flanking homology (*URA3* sequences) that can be used for repair by SSA yielding Δproduct (the grey area within *URA3::*Ty is a naturally disrupting Ty element). (B) The *ade2*::*arg4*-vde reporter cassette is hemizygous, inserted into one *ADE2* locus. The opposite Chromosome XV has an internal deletion in the *ADE2* locus. Resection of approximately 3.0 kb to both the left and right will uncover the proximal *URA3* repeated DNA sequences. An SSA event between these yields Δproximal. Further resection to 4.5 kb and approximately 7.0 kb to the left and right will uncover the *ADE2* repeated sequences; SSA between them yields Δdistal. Repair of VDE-DSB2 by gene conversion is unlikely as homology with the *ade2*Δ chromosome is over 3 kb and 7 kb away, on the left and right sides, respectively, leading to long nonhomologous 3′ ends.

Previous studies have shown that a DSB created by VDE (described as VDE-DSB1 below) can be repaired either through an interhomolog gene conversion event, or by using direct repeats that flank the DSB site, causing deletion of intervening sequences and creating “Δproduct” ([31]; Figure 1A). Both sister chromatids containing the VDE-recognition sequence are usually cleaved during meiosis, and tetrad analysis revealed both chromatids are frequently repaired to Δproduct, an outcome most compatible with repair by SSA [31]. The proportion of VDE-DSB1s repaired by interhomolog gene conversion versus repair using flanking repeated sequences, creating Δproduct, varied significantly between cells with mutations that either inhibited Spo11-DSB formation or single-strand resection at Spo11-DSBs. The former caused an increase in both the rate of VDE-DSB1 repair and the proportion of Δproduct, whereas the latter slowed repair and reduced the proportion of Δproduct. To further determine how the presence of Spo11-DSBs can influence VDE-DSB repair in trans, we have now investigated two VDE-DSB sites in *dmc1*Δ meiotic cells. Mutating *DMC1* allows efficient Spo11-DSB formation and efficient single-strand resection, but Spo11-DSBs fail to repair due to an inability to form interhomolog joint molecules. Thus, in *dmc1Δ* cells the VDE-DSBs are in the context of multiple hyper-resected Spo11-DSBs.

In addition to the published reporter construct [31], we used a second VDE-DSB reporter construct, inserted on Chromosome XV. In this cassette there are two pairs of nested direct repeats flanking the VDE cleavage site (VDE-DSB2). The proximal repeats contain *URA3* sequence; the distal repeats contain *ADE2* sequence (Figure 1B). The second Chromosome XV has a 645-bp deletion from the *ADE2* locus. No homology to repair VDE-DSB2 exists on the homologous chromosome for approximately 4 kb to the left and 7 kb to the right of VDE-DSB2, forcing DSB repair events to proceed through an intrachromosomal route, which we show is SSA.

### The VDE-DSB1 Accumulates in Cells Lacking Dmc1 Function

In wild-type cells, VDE-DSB1 is repaired by both interhomolog gene conversion and SSA. We considered that knocking out *DMC1* could have very different influences on VDE-DSB repair. On the one hand, RecA type strand invasion function is not required for SSA, and so in *dmc1*Δ cells there could have been efficient VDE-DSB repair by SSA, at the expense of gene conversion. On the other hand, in *dmc1*Δ cells the nucleus accumulates around 200 resecting Spo11-DSBs and hundreds of kilobases of ssDNA [5]. This might create a competition for resources (such as resection proteins or RPA) and therefore compromise VDE-DSB repair.

**Figure 2:**
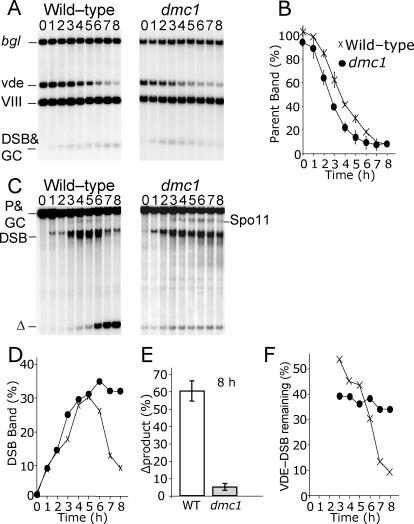
Repair of VDE-DSB Is Inhibited in *dmc1* Cells (A) DNA from 0 h to 8 h of meiotic culture was digested with EcoRV and BglII and probed close to the VDE-DSB1 site to create a DNA fragment of unique size containing uncut parental *arg4*-vde DNA (bgl = parental *arg4*-bgl chromatids plus gene conversion products creating further *arg4-*bgl*;* vde = parental *arg4*-vde chromatids; VIII = *arg4*-nsp;bgl chromatids in both natural Chromosome VIII loci; DSB&GC= VDE-DSB1 molecules plus gene conversion products creating *ARG4*). (B) Quantification of the parental *arg4*-vde band normalized to 50% of signal in band VIII. Diminution of the *arg4*-vde band is a consequence of VDE-DSB1 formation, which occurs at similar rates in wild-type and *dmc1* cells. (C) DNA was digested with SpeI and probed distal to the *URA3* locus to isolate DNA fragments of unique size containing VDE-DSB1 molecules and Δproduct (P&GC = parental *arg4*-vde and *arg4-*bgl chromatids plus gene conversion products; DSB = chromatids with VDE-DSB1; Δ = Δproduct; Spo11 = natural Spo11-DSB site close to the *arg4* insert). (D) Quantification of VDE-DSB1 signal expressed as a proportion of *arg4*-vde-containing chromatids, symbols as in (B). (E) Quantification of Δproduct expressed as a proportion of parental *arg4*-vde chromatids. (F) Quantification of VDE-DSB1 signal expressed as a proportion of cumulative parental *arg4*-vde chromatids that have received a VDE-DSB1, symbols as in (B).

Using Southern analysis that isolates a parental *arg4*-vde DNA fragment from all other *arg4*-containing species, we determined that mutating *DMC1* had no significant effect on the efficiency of forming VDE-DSB1 (Figure 2A and 2B). By contrast, mutating *DMC1* causes a major delay in turnover of the VDE-DSB1 DNA (Figure 2C and 2D). In wild-type cells, the VDE-DSB1 signal accumulates up to 5 h after induction of meiosis and gradually disappears as the rate of DSB repair outpaces residual DSB formation. The VDE-DSB1 signal accumulated in *dmc1*Δ cells similarly to wild type up to 4 h after induction of meiosis, but did not diminish. Consistent with the observed accumulation of VDE-DSB1 molecules in *dmc1*Δ cells, very little Δproduct was created compared to wild-type cells (Figure 2E). As there is no significant difference in the kinetics of VDE-DSB1 formation in the different strains, the observed variation in VDE-DSB1 levels must be due to a difference in repair.

In *dmc1*Δ cells, the amount of VDE-DSB1 DNA detected on Southern blots was maintained from 4 h to 8 h at a steady level representing approximately 35% of chromatids (Figure 2F). However, by 8 h of meiosis, less than 10% of chromatids were detectable in the Δproduct band, even though approximately 90% of chromatids had been broken (Figure 2B). The discrepancy between these values suggested that standard Southern analysis fails to detect some VDE-DSB1 DNA. This could be due to extensive single-strand resection, which is known to cause Spo11-DSB bands to become diffuse when DNA is extracted from *dmc1*Δ cells [5].

### Accumulated VDE-DSBs Are Resected

**Figure 3:**
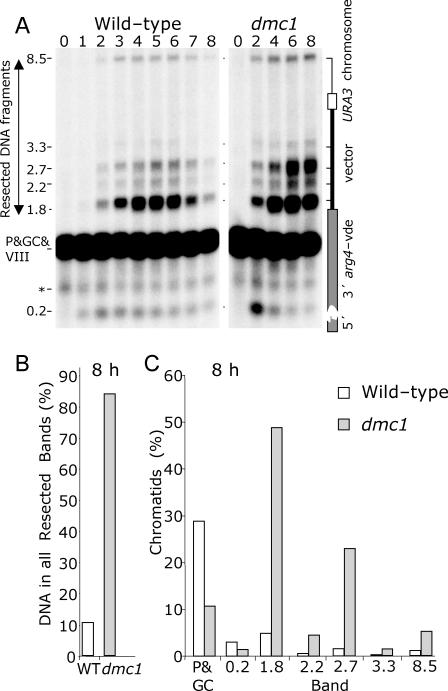
Most Broken *arg4*-vde-Containing Chromatids in *dmc1* Cells Remain Unrepaired in a Resected State (A) DNA from 0 h to 8 h of meiotic culture DNA was digested with HaeII and fractionated by alkaline denaturing gel electrophoresis. Bands have increasing molecular weight as the restriction sites are progressively destroyed by resection. The numerals to the left indicate distance from the VDE-DSB1 site to an HaeII cut-site, and represent the maximum extent of resection for molecules in the respective bands (* = nonspecific band; other labels are as described in Figure 2; a maximum of 1/4 of the signal in the P&GC&VIII band represents the parental *arg4*-vde fragment; full details in Materials and Methods). (B) Quantification of the resected bands totaled after 8 h of meiosis expressed as a proportion of parental *arg4*-vde chromatids. In *dmc1*Δ cells practically all VDE-DSB1s created remain in a resected state. (C) Quantification of individual resected bands after 8 h of meiosis expressed as a proportion of parental *arg4*-vde chromatids. Accumulation of resected molecules is punctuated in *dmc1*Δ cells indicating likely pause sites through which resection is nonprocessive. (P&GC represents remaining parental chromatids plus gene conversion product; the numbers refer to band sizes).

To test whether or not the VDE-DSB1 band was underrepresented due to diffuse smearing, we compared the amount of resected DNA that accumulates in wild-type and *dmc1*Δ cells using a loss-of-restriction site assay. This assay resolves DNA under denaturing conditions and quantitatively detects resected molecules as discrete bands by using a strand-specific probe (Figure 3A). In the wild-type culture after 8 h, approximately 11% of *arg4*-vde chromatids were detected in bands representing VDE-DSB1 resected DNA (Figure 3B). By contrast, 8 h after induction of meiosis in the *dmc1*Δ culture, 84% of VDE-DSB1 DNA was detected in the resection bands (Figure 3B). Since 90% of *arg4*-vde chromatids are cut by VDE by 8 h meiosis (Figure 2B), virtually all VDE-DSB1s in *dmc1*Δ cells persist in a resected, unrepaired state.

Persistence of resected DNA around VDE-DSB1 is highly reminiscent of the behavior of Spo11-DSBs in *dmc1*Δ cells. We found most of the resected DNA appears in two apparent pause sites for resection (Figure 3A and 3C). One pause site, with resection up to 1.8 kb, is also present in earlier time points for wild-type cells, and in *dmc1*Δ cells contains approximately 49% of VDE-DSB1 chromatids at 8 h (Figure 3C). The second pause site, with up to 2.7 kb of resection, appears to be a major block to further processing in *dmc1*Δ cells with 23% of VDE-DSB1 chromatids collecting there by 8 h (Figure 3C). Repair of the VDE-DSB1 by SSA requires that resection extends as far as both flanking copies of the repeated sequence. To uncover the repeat sequence on the right side of VDE-DSB1 requires over 6 kb of resection. Thus, the difficulty in repairing the VDE-DSB1 by SSA may be caused by the inability to create sufficiently extensive resection tracts in the *dmc1*Δ cells.

Another important feature of this analysis is a 5-fold increase in the band representing resection between 3.3 kb and 8.5 kb in *dmc1*Δ cells, compared to wild-type (Figure 3A and 3C). This band is barely visible in wild-type cells, presumably due to rapid repair by SSA of DSBs with long resection tracts. Thus, even when there is sufficient resection for SSA, repair is inhibited in *dmc1*Δ cells.

### Repair of VDE-DSB1 Is Not Dependent on Meiotic Progression

**Figure 4:**
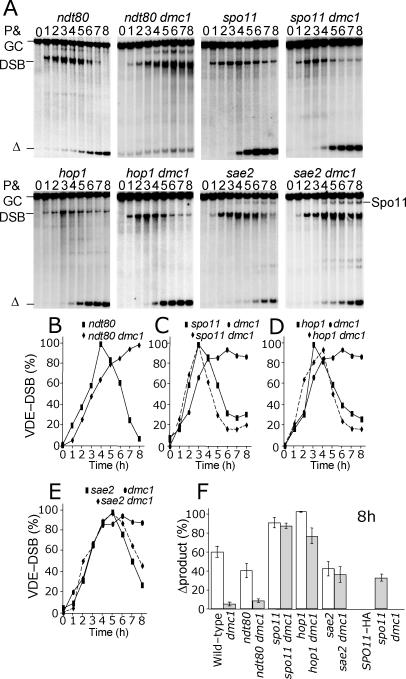
Meiotic Prophase Arrest Does Not Inhibit VDE-DSB1 Repair, but the Quantity and State of Spo11-DSBs Affects the Need for Dmc1 In all cases *spo11* refers to *spo11*-Y135F-HA3His6. (A) DNA from 0 h to 8 h of meiotic culture was digested and displayed as described in Figure 2C. (B) Cells do not require Ndt80 function to turnover the VDE-DSB in *DMC1* cells, and *dmc1*Δ is epistatic to *ndt80* for VDE-DSB1 repair. The repair defect imposed by loss of Dmc1 function is fully rescued by (C and D) removing all or most Spo11-DSB formation in either *spo11* or *hop1* mutant cells and by (E) inhibiting resection at Spo11-DSBs by mutating *SAE2*. (F) The epistatic relationships between the various mutations and *dmc1* are also displayed by quantification of Δproduct after 8 h of meiotic culture, expressed as a proportion of parental *arg4*-vde chromatids. Wild type and *dmc1* are shown for comparison. Reducing the quantity of Spo11-DSBs to 50% of wild-type levels using a strain heterozygous for different *SPO11* alleles leads to partial relief of Dmc1 dependence for VDE-DSB1 repair.

*dmc1*Δ cells arrest meiosis prior to exit from prophase, due to checkpoint-induced inactivation of the Ndt80 transcription factor [7,33–35]. To test the possibility that this arrest causes inhibition of resection and SSA, we repeated the assay in cells lacking *NDT80* (Figure 4).

The kinetics of VDE-DSB1 induction and repair were similar in wild type and *ndt80* meioses (Figure 4A and 4B). While *ndt80 dmc1*Δ mutants displayed the same defects in VDE-DSB1 repair as *dmc1*Δ cells, *ndt80* cells exhibited amounts of Δproduct comparable to wild type (Figure 4F). Thus, the meiotic prophase arrest imposed by *dmc1*Δ is unlikely to be the cause of the inefficient VDE-DSB1 repair.

### Mutants That Reduce the Cellular Load of Resected Spo11-DSBs Restore SSA of VDE-DSBs in *dmc1*Δ Cells

We next considered the possibility that VDE-DSB repair is reduced in *dmc1*Δ cells because of the large quantity of ssDNA produced by the resection of unrepaired Spo11-DSBs. To test this, we measured the efficiency of repairing VDE-DSB1 in *dmc1*Δ cells with few Spo11-DSBs (*hop1*Δ), completely lacking Spo11-DSBs (*spo11*-Y135F-HA3His6 [36], referred to as *spo11*, forthwith), and in *sae2*Δ cells with wild-type levels of Spo11-DSBs but no single-stranded resection (Figure 4A and 4C–4F).

We previously reported that mutation of *HOP1*, *SPO11*, or *SAE2* alters the relative proportion of VDE-DSB1 molecules that repair using the flanking repeats versus repair by gene conversion. In that study, and here, we found that mutating *HOP1* or *SPO11* increased both the speed of repair and the proportion of VDE-DSB1 that repaired using flanking homology [31] (Figure 4A, 4C, 4D, and 4F). In *sae2*Δ cells, VDE-DSB1 repair is slower and the proportion of molecules that repair using flanking repeats is reduced, probably due to a direct role of Sae2 on resection, even at DSBs lacking covalently bound protein [32] (Figure 4A, 4E, and 4F) .

We now report that all the double mutants, *spo11 dmc1*Δ, *hop1*Δ *dmc1*Δ, and *sae2*Δ *dmc1*Δ behave almost the same as their *DMC1* single mutant counterparts with respect to the kinetics and efficiency of VDE-DSB1 repair (Figure 4A and 4C–4F). In other words, the requirement for Dmc1 to repair a VDE-DSB using flanking homology is relieved by mutations that reduce the large quantity of resected Spo11-DSBs and ssDNA usually seen in *dmc1*Δ meiosis.

### Lowering the Quantity of Resected Spo11-DSBs Reduces the Requirement for Dmc1 to Repair VDE-DSB1 by SSA

We next checked if intermediate levels of VDE-DSB repair could be achieved in *dmc1*Δ cells when intermediate levels of ssDNA accumulate in the nucleus. The number of Spo11-DSBs was reduced by using a heterozygous diploid expressing the hypomorphic *SPO11*-HA3His6 allele and the null allele, *spo11*-Y135F-HA3His6 [36]. This combination of *SPO11* alleles is reported to reduce recombination and Spo11-DSB levels to approximately 50% of wild type [37]. We confirmed this to be the case in *dmc1*Δ cells by measuring DSB formation at the *ARE1* hotspot (unpublished data). This reduction in Spo11-DSB formation increased the VDE-DSB1 repair efficiency in *dmc1*Δ cells approximately 6-fold (Figure 4F), supporting the contention that inefficient SSA repair of VDE-DSBs in *dmc1*Δ cells is related to accumulation of resecting Spo11-DSBs.

### Dependency on Dmc1 for VDE-DSB1 Repair Does Not Reflect the Requirement for an Unexpected DNA Strand Invasion Event

**Figure 5:**
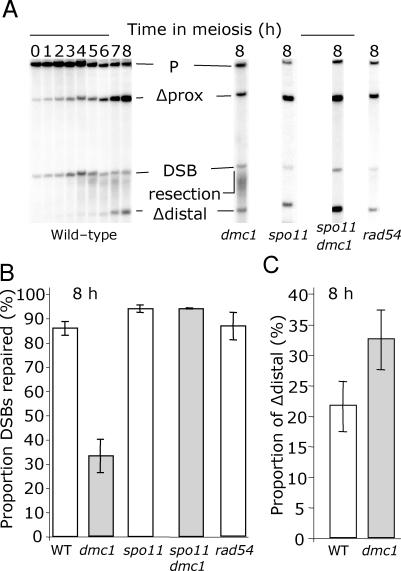
Southern Analysis of the VDE-DSB2 Cassette in a Hemizygous Context In all cases *spo11* refers to *spo11*-Y135F-HA3His6. (A) DNA from wild-type and mutant cells, as indicated, was digested with SpeI, which isolates unique fragments representing (P) the uncut parent *arg4*-vde chromatid, (Δprox) the product of SSA between *URA3* proximal repeated sequences, (DSB) VDE-DSB2 molecules, and (Δdistal) the product of SSA between *ADE2* distal repeated sequences. Cutting by VDE is less efficient in this assay, as indicated by the remaining parental DNA. In all but the *dmc1*Δ mutant cells, little VDE-DSB DNA is visible by 8 h. The amount of product accumulating is significantly reduced in *dmc1*Δ cells in which the VDE-DSB2 becomes smeared due to prolonged resection. (B) The total amount of repair product (sum of Δdistal and Δproximal) visible has been measured as a proportion of *arg4*-vde-containing chromatids that have received a VDE-DSB by 8 h in meiosis (details in Materials and Methods). (C) The quantity of Δdistal expressed as a proportion of sum of total repair product gives an indication of how often repaired molecules used the longer resection tract.

Theoretically, VDE-DSB1 could create the Δproduct following an unequal strand exchange event with the homologous chromosome, which could be Dmc1-dependent in meiosis. It was important therefore to test repair of a VDE-DSB in a context that ruled out the possibility of Dmc1-dependent interhomolog repair. To test this, we used a second reporter cassette that contains the VDE cleavage site (VDE-DSB2) inserted on Chromosome XV between two pairs of nested direct repeats (Figure 1B). Repair of VDE-DSB2 by strand invasion with the homolog is precluded by large tracts of heterology present in the hemizygous reporter insert. Repair of VDE-DSB2 was also reduced by loss of *DMC1*, although not to the same extent as VDE-DSB1 (Figure 5A and 5B). By 8 h in the *dmc1*Δ culture, approximately 33% of VDE-DSB2 broken chromatids had repaired compared to 86% in wild type (Figure 5B). The repair level did not increase in *dmc1*Δ cells by 12 h (unpublished data). As for VDE-DSB1, repair of VDE-DSB2 was increased in *dmc1*Δ cells when the DSB forming function of Spo11 was removed (Figure 5B).

By mutating the *RAD54* gene, which is important for strand exchange between sister chromatids, we confirmed our expectation that repair of VDE-DSB2 using flanking repeated sequences was not by unequal intersister repair [6,9,38,39]. For *rad54* cells, there was a slight delay in meiosis indicated by delayed induction of VDE-DSB2 and late onset of the first division (unpublished data). Despite this delay, 87% of broken chromatids molecules were repaired by 8 h in *rad54* meiosis (Figure 5B). Because VDE-DSB2 repair was as efficient in *rad54* cells as in wild type, we conclude that repair using the flanking repeats does not require either a homolog or Rad54. In other words, VDE-DSB2 repair occurs by SSA, rather than via a mechanism that requires DNA strand invasion of a donor duplex.

Evidence that VDE-DSBs cannot repair using the sister chromatid in meiosis is supported by analysis of a *his4*-vde allele in a hybrid strain containing a Saccharomyces cerevisiae Chromosome III opposite a Saccharomyces carlsbergensis Chromosome III. Due to sequence divergence between the two chromosomes, interhomolog gene conversion is prohibited [40–42]. Since no repeats flank the *his4* VDE-DSB, the only possible route to homologous repair is through invasion of the sister chromatid. However, all tetrads are two-spore viable due to loss of the *his4*-vde Chromosome III, indicating that intersister strand invasion and repair does not happen (K. Tittcomb and A. S. H. Goldman, unpublished data).

### Resection at VDE-DSB2 Can Be Extensive in *dmc1*Δ Cells

Our analysis of resection intermediates at VDE-DSB1 suggested that DSB repair might be inhibited in *dmc1*Δ cells because of a failure to perform sufficient extensive ssDNA resection (Figure 4). Because VDE-DSB2 is flanked by nested direct repeats (Figure 1B), this site can be used to estimate how much resection had occurred in the repaired molecules. Repair by SSA using the proximal repeats (Δprox) creates a 5 kb deletion, detected on Southern blots as a 10 kb band. Repair using distal repeats (Δdistal) creates a 10.5 kb deletion and a 4.5 kb band (Figure 5A). By comparing the relative proportion of these repaired products, it is possible to estimate the proportion of molecules that repaired with longer resection tracts (Figure 5C). In wild-type cells, approximately 22% of repaired VDE-DSB2 molecules used the distal repeats. In *dmc1*Δ cells, the distal repeats were used more frequently, with 33% of repaired molecules being of the Δdistal type. Thus, the reduction in resection in *dmc1*Δ cells is not uniform across the population of cells, possibly reflecting a stochastic inhibition created by competition for resources.

### Rfa1, a Component of the ssDNA Binding Complex RPA, Has Limited Access to the Repeated Sequences Flanking VDE-DSB1 in *dmc1*Δ Cells

The negative correlation between ability to repair the VDE-DSB by SSA and the number of DSBs undergoing extensive resection could reflect inability of ssDNA binding proteins to access homologous sequences flanking the VDE-DSBs. Failure of ssDNA binding proteins to bind DNA could result from a combination of reduced resection and ssDNA binding proteins being in limited supply at the VDE-DSBs, because they are sequestered to the long ssDNA tracts that accumulate at the many Spo11-DSBs present in the cell.

**Figure 6:**
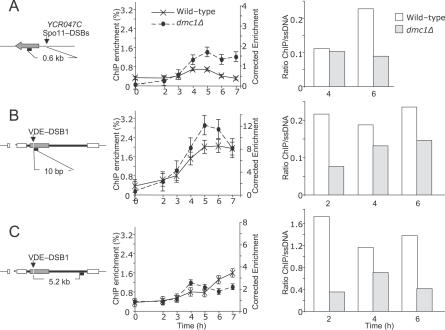
ChIP Reveals That RPA Is a Limiting Factor in *dmc1Δ* Cells (A–C) DNA associated with immunoprecipitated Rfa1, a component of RPA, was amplified and quantified by qPCR. The values are averages from duplicate experiments. Each diagram shows the distance of PCR primers from the relevant DSBs; grey arrow represents *YCR047C*, the cassette containing the VDE-DSB1 site is as in Figure 1A, the small black bars represent the position of the PCR products at the indicated distance from the DSB site. The left axis of the graphs show ChIP enrichment (i.e., ChIP signal relative to input signal; %); on the right axis the values have been corrected to account for overrepresentation of substrate in the input compared to the proportion of probed chromatids that can receive a DSB (see Materials and Methods). The bar charts show the ratio of corrected ChIP enrichment values to proportion of chromatids that are expected to contain ssDNA in the PCR-amplified region. In each bar graph the ratio of corrected ChIP enrichment to estimated levels of ssDNA are always higher in wild type compared to *dmc1*Δ, showing that the immunoprecipitation of Rfa1 is more efficient in wild-type cells. The difference in ChIP efficiencies for wild-type and *dmc1*Δ cells is greatest for the site far from VDE-DSB1, implying that as resection proceeds it becomes more difficult to compete for Rfa1, which may be stably bound to ssDNA created earlier, close to DSB sites. (A) Close to a Spo11-DSB hotspot, the ChIP enrichment decreases with repair in wild-type cells but not in *dmc1*Δ cells. (B) Close to VDE-DSB1, the ChIP enrichment in *dmc1*Δ cells is no higher than in wild-type, even though by 7 h around 80% of *arg4*-vde-containing chromatids are in a resected state (see Figures 2 and 3) and (C). Distant from VDE-DSB1, but close to a flanking repeated sequence used for SSA, again the ChIP enrichment in *dmc1*Δ cells is no higher than in wild type, even though significant amounts of DNA accumulate with resection beyond this point (see Figure 3).

The ssDNA binding protein complex, RPA (also known as RF-A in yeast), is required to remove secondary structure in ssDNA and to recruit recombination proteins such as Rad52 during homologous recombination [43]. Using chromatin immunoprecipitation (ChIP), we compared (in wild-type and *dmc1*Δ cells) the association of an RPA component (Rfa1) to DNA coupled with a Spo11-DSB hotspot (YCR047C/BUD23 ORF, [44]) or with VDE-DSB1 (Figure 6A–6C). Close to the *BUD23* Spo11-DSB hotspot, a small amount of DNA is enriched from wild-type cells by ChIP, the changing levels through time reflect the kinetics of appearance and disappearance of Spo11-DSBs at this site (Figure 6A). In *dmc1*Δ cells, the Rfa1 signal is not reduced in later time points because Spo11-DSBs are not repaired. Normally Spo11-DSBs are formed up to 6 h after induction of meiosis. Interestingly, Rfa1 does not accumulate to higher levels after 4 h as more Spo11-DSBs are created, perhaps because as Rfa1 becomes limiting it is competed away from a proportion of the sites close to the Spo11-DSB.

We have already shown that almost all VDE-DSBs created (in close to 100% of cells; Figure 2B) in *dmc1*Δ remain broken and in a resected state throughout the time course (Figure 3). We reasoned that if Rfa1 coats all ssDNA in *dmc1*Δ cells, the Rfa1 ChIP signal should be higher in *dmc1*Δ cells versus wild type.

Using two different primer sets, one close to the VDE-DSB1 (Figure 6B) and one close to a flanking repeated sequence (Figure 6C), we found that the ChIP signal was not significantly enhanced in *dmc1*Δ cells close to the VDE-DSB and is slightly reduced far from the VDE-DSB1. Taken together, these results support the view that when Spo11-DSBs remain in a resected state, Rfa1 does not accumulate at specific ssDNA sites, even though the quantity of ssDNA is demonstrably increasing (Figure 3). Thus, for those cells, in which resection does uncover the repeated sequences flanking the VDE-DSB1, there may not be sufficient RPA to mediate repair by SSA.

We noted that the ChIP signal is not significantly reduced by 7 h in wild-type cells, when a large proportion of VDE-DSB1s are repaired. In part this is probably because 40% of the peak numbers of VDE-DSB1s are still present at this time (Figure 2D). It is also possible that RPA is not removed as rapidly from the DNA following SSA as it would be following classical homologous recombination.

## Discussion

In budding yeast, repair of DSBs induced by VDE is a natural process, which propagates VDE-containing genetic elements from one chromosome to another during meiosis [45]. Previous studies have shown the timing of DSB induction by VDE and the mechanisms of repair parallel closely those of Spo11-induced recombination [28,31]. Deleting *DMC1* is expected to prevent gene conversion events at VDE-DSB sites, but in addition we have found it also inhibits repair by SSA, a process that in mitosis neither requires strand invasion nor the RecA ortholog Rad51 [17].

The failure in VDE-DSB repair in *dmc1*Δ mutant cells can be relieved by mutations that eliminate Spo11-DSBs, reduce their frequency, or prevent their resection. Furthermore, in *dmc1*Δ cells resection at VDE-DSBs is reduced, and Rfa1 (a component of the ssDNA binding complex, RPA) is unable to access the repeated sequences flanking the VDE-DSB sites.

### Repair Proteins Are a Limiting Factor

**Figure 7:**
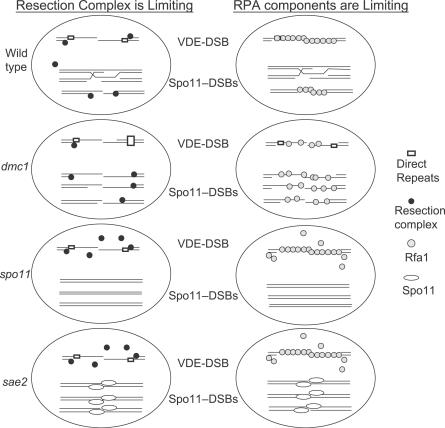
Limited Availability of DNA Repair Proteins Explains the Requirement of Dmc1 for SSA at VDE-DSBs In wild-type meiosis there is sufficient resection complex and Rfa1 to create and bind to long tracts of ssDNA at the VDE-DSB so that SSA is possible. In part, the ready supply of such proteins is likely created by the asynchronous nature of Spo11-DSB formation and repair in the nucleus, thus when some Spo11-DSBs are using these proteins others may have moved to a biochemical step that allows their release. In *dmc1*Δ cells, so much resection complex and Rfa1 is sequestered to multiple unprepared Spo11-DSBs that insufficient resection complex is available to create long resection tracts at the VDE-DSB; or in cases where long resection tracts appear there is not enough free Rfa1 to bind the repeated sequences. Mutating either *SPO11* or *SAE2* relaxes the demand on both resection proteins and ssDNA binding proteins such that resection and repair of the VDE-DSB is no longer limiting.

Losing Dmc1 function has an enormous impact on the normal balance of DNA transactions taking place in the meiotic nucleus. Spo11-DSBs are formed with wild-type kinetics, but breaks remain unrepaired and accumulate genome-wide, so that at later time points each cell would contain about 200 lesions [5]. Under these conditions, resection continues for many hundreds or thousands of nucleotides further than normal, and therefore the demand for both resection complex and RPA is likely to be extremely high and critical (Figure 7).

The data from two VDE-DSB-containing cassettes support the idea that either resection complex or ssDNA binding proteins can become limiting. Insufficient access to resection complex could reduce resection below the lengths needed to render flanking repeats single-stranded. Insufficient RPA to coat long resection tracts could prevent SSA even if resection has uncovered the repeated sequences. Whether a protein complex becomes limiting at a particular DSB may be stochastic, though genome location and immediate environment may also be important. This would explain why at VDE-DSB1 resection appears extremely limited, but less so at VDE-DSB2, which also repairs poorly but more efficiently than VDE-DSB1.

The coordinate induction and repair of DNA damage is a vital part of the meiotic developmental pathway. Ensuring that the many proteins required for DSB repair are in appropriate supply and in active form is a major task for the cell. Spo11-DSB formation is temporally linked to replication, with DSBs appearing about 1.5 h after replication has passed through [46]. Thus, like replication, the formation of Spo11-DSBs across the genome is asynchronous. In between Spo11-DSB formation and repair, Spo11-DSBs are processed by 5′ to 3′ resection. Resulting ssDNA subsequently forms joint molecules with the homologous chromosome, and DSBs are repaired 1–1.5 h after Spo11-DSB formation [47,48]. It is reasonable to assume that by this time resection would have ceased and ssDNA-binding complexes such as RPA and Dmc1/Rad51 may be liberated for reuse. Since a large number of such molecules are required at every DSB, the asynchronous induction of recombination may have evolved to spread the workload of proteins, which otherwise would have to be produced at levels that might result in pleiotropic negative consequences.

There is precedence for proteins involved in nucleic acid metabolism being in limiting supply to avoid pleiotropic effects from over abundance. Activity of ribonucleotide reductases (RNRs) is tightly controlled to ensure nucleotide pools are sustained at appropriate levels. Control is maintained at the transcriptional level and by an inhibitor protein, Sml1 ([49] and within)*.* The Mec1 DNA damage response pathway, acting through Rad53 and Dun1, regulates both controls. DNA damage increases transcription of RNR genes and phosphorylates Sml1 causing its inactivation [50,51]. Regulation of active RNR protein supply is so tight that mutations in the Mec1 kinase cascade cause *SML1* expression to become toxic, due to insufficiency of nucleotides needed for routine DNA repair [50,52,53]. Stringent control of RNRs probably reflects the fact that overabundance of nucleotides can be mutagenic due to increased risk of misincorporation.

Our data suggest that ssDNA binding proteins must also be kept in limiting supply. This suggestion fits well with the fact that the transcription regulation of RPA components is linked to the cell cycle, peaking at the G1/S boundary [54]. Furthermore, reports on in vitro DNA binding and activity of Rad52 and Rad51 indicate that RPA at low concentrations promotes strand exchange, yet at high concentrations it limits access of repair proteins to the DNA [55–57].

### Limited Supply of Repair Proteins Could Have Wide-Ranging Impact on Regulating Meiotic Processes

That Rfa1 can become a limiting factor in an experimental situation raises the possibility that in wild-type cells perturbations of repair efficiency or replication could have indirect effects on other repair/replication processes. Such effects can be at sites other than the original lesion. For example, a level of DNA damage similar to that experienced by meiotic cells might perturb DNA replication, because damage repair sequesters protein factors such as RPA that are also critical for replication.

One reason for limiting the number of Spo11-DSBs in yeast meiosis could be to ensure that a safe and sustainable balance can be reached between repair protein supply and demand within a suitable time frame. Studies on mammalian and yeast vegetative cells support the view that homologous recombination is extremely sensitive to protein supply, as overexpression of Rad52 epistasis group proteins can inhibit repair of an experimentally induced DSB [58,59]. Consistent with the notion that protein supply and demand is finely balanced in meiosis, moderately hypomorphic mutants of DSB repair genes such as MRE11, NBS1, or RAD51C have significantly reduced fertility in mice due to inefficient DSB repair [60,61]. Similarly in yeast meiosis, deleting one RecA ortholog, *DMC1*, causes a complete block in DSB repair that can be largely rescued by either overexpressing or releasing inhibition of *RAD51* [62,63].

It was recently shown that synchrony of the first meiotic division could be influenced by temporary chemical inhibition of a specific Cdc7 activity required for induction of Spo11-DSBs [64]. Nearly half of the population underwent the first meiotic division sometime between 3 h and 4 h after inhibitor wash out, with no loss of viability. This might seem to counter our argument that repair proteins are limiting. But across the population at the single Spo11-DSB analyzed, break induction was still spread over some hours. Thus, while the Wan et al. data [64] demonstrate some improved synchrony of the first division across a population of cells, it does not directly address the synchrony of DSB formation within each cell. Further analysis of highly synchronized populations, in which the time of inducing Spo11-DSBs and their life spans is well defined, will help to clarify the time limits within which Spo11-DSB formation must be limited to avoid problems of repair protein supply.

Limiting protein supply could serve a useful function other than protecting against ill effects of overproduction, such as to direct the proportion of events that take one biochemical pathway rather than another [58]. One possible example of this comes from maize, which produces more than 20-fold excess of DSBs during meiosis compared to the known crossover frequencies [65]. These DSBs are identified cytologically as Rad51 foci that often appear as opposite pairs on homologous chromosomes and may be used as pairing sites [65,66]. What prevents a much higher proportion of DSBs in maize meiosis from becoming crossovers is unknown. Perhaps a protein required for crossover-associated repair is in limited supply, so the majority of DSBs can serve a function other than forming crossovers.

The impact single mutations have on biochemistry, nucleus-wide, is rarely known. This study highlights the fact that indirect effects can easily arise from changing the balance of protein supply and substrate in the nucleus. In nature, tight control of protein supply may be essential for avoiding pleiotropic effects from oversupply, or for limiting the number of events passing down a specific pathway. In the laboratory, phenotypes ascribed to mutations may often not be caused by a direct biochemical impact of mutating a gene, but may be due to broader biochemical imbalances created across the nucleus. Genes involved in DNA metabolism may be particularly likely to cause pleiotropic phenotypes. Mutations altering the amount of damage present in a nucleus are certainly prone to pleiotropic effects that are worth serious consideration when defining protein function.

## Materials and Methods

### Media, genetic methods, and strains.

Diploid yeast strains of the SK1 background relevant genotypes are listed in Table S1.

The *ura3*::*arg4*-vde cassette (Figure 1A) was created as described previously in [31]. The *ade2*::*arg4*-vde reporter cassette (Figure 1B) was created by transforming parent strains with pAG408, a derivative of pBR322 based pAG137 [31]. The *URA3* of pAG137 was modified by a 5′ (−17 to +129) deletion between restriction sites SdaI to XcmI. A functional *URA3* (HindIII fragment) was inserted into an NruI site in the PBR322 backbone. A section of the *ADE2* ORF (+250 to +1695) generated by PCR from yeast genomic DNA was inserted into the pBR322 EcoRI site and used for integration into the yeast genome following linearization at the AflII site.

Relevant mutant strains containing either the *ura3*::*arg4*-vde or *ade2*::*arg4*-vde reporter cassettes were made by mating and dissection with appropriate SK1 haploids. The source of the *spo11*-Y135F-HA3His6; *hop1*; *sae2*Δ; and *ndt80* haploids is reported in [31].

For the *ura3::arg4-*vde reporter construct, *dmc1* disruption was made in pAG64 using primers 5′-gccattctatgtctgatcccgg-3′ and 5′-tcgcttagttcacctctaccgc-3′ to amplify a 1,466-bp region of the *dmc1* locus from haploid SK1 genomic DNA. MfeI-cleaved PCR product was ligated into EcoRI-linearized pUC19. A 2.2 kb *ADE2-*containing BglII fragment of pAG52 ( pMJ412 from M. Lichten) was ligated at the BglII site located 90 bp inside the *DMC1* ORF. For the *ade::arg4-*vde reporter cassett, *dmc1::ARG4* was obtained from D. Bishop in SK1 and was crossed into our experimental strains. The *rad54*Δ mutation was obtained from D. Bishop and transformed into relevant strains.

### DNA isolation and Southern blot analysis.

40 ml samples of culture were removed at hourly intervals and processed for storage and DNA isolation according to Allers and Lichten (2001); hexamine cobalt (III) chloride was excluded from solutions.

Restriction endonuclease–digested DNA was separated under native conditions or denaturing conditions. Separated DNA was blotted to Zetaprobe membrane (Bio-Rad) under denaturing conditions with a Vacugene-XL system. Analysis of VDE-DSB1 Δproduct and resection intermediates was undertaken as described [31]. For VDE-DSB2 the probe used to display SpeI- digested DNA after native separation is specific to Chromosome XV coordinates 566120–566811.

### Quantification and calculations for Southern analyses.

Quantification was as described [31]; briefly, for VDE-DSB1 the amount of DNA in the VDE-DSB1 band and Δproduct were determined as a proportion of *arg4*-vde-containing chromatids by dividing the signal in each band by half of total signal (which represents both *arg4*-vde and *arg4*-bgl chromatids). For the denaturing gels, the amount of signal attributable to chromatids that contained parental *arg4*-vde insert was calculated taking into account the presence of signal from six other chromatids (two chromatids with *arg4*-bgl on the homologous Chromosome V and four chromatids with *arg4*-nsp,bgl at the natural *ARG4* locus on both Chromosome VIII homologues) and the fact that chromatids repairing to Δproduct do not contribute signal.

If;

Δ_r_ = Recorded proportion of *arg4*-vde chromatids repaired by SSA

S_r_ = Signal recorded in lane on denaturing gel

Δ_m_ = Δproduct missing from lane on denaturing gel

R_r_ = Resection band recorded signal on denaturing gel

1/4 of total possible signal comes from *arg4*-vde chromatids

Then

Δ_m_ = 0.25Δ_r_S_r_/(1−0.25Δ_r_)

Therefore signal calculated in lane attributable to *arg4*-vde chromatids;

S_c_ = 0.25(Δ_m_+S_r_)

Then proportion of *arg4*-vde chromatids in each resection band;

R_c_ = R_r_/S_c_

For VDE-DSB2 gels, the DNA in each band was quantified.

If

T = Total signal in lane

P = Signal in parent band

Δprox = signal in Δprox band

Δdistal = signal in Δdistal band

Then DNA repaired, as a proportion of breaks made equals (Δprox + Δdistal)/(T − P) and the proportion of DNA repaired to Δdistal equals Δdistal/(Δprox + Δdistal).

### Rfa1 ChIP.

20 ml cells (4 × 10^9^ cells) were treated with 1% fresh formaldehyde for 15 min at room temperature and 125 mM glycine for 5 min. ChIP was performed as described [67] using magnetic protein G beads (Dynal) and a polyclonal rabbit anti-Rfa1 antibody (supplied by S. Brill). Enrichment of DNA bound by RPA was estimated by quantitative PCR using an Applied Biosystems 7500 Real-Time PCR system with 0.4 μM primers, SYBR Green PCR master mix (Applied Biosystems), and the PCR program: 95 °C for 15 s; 60 °C for 1 min; 40 cycles. The following primers were used: *YCR047c*; 5′-TATGTCGTCCACCTGGTCGTCG-3′ and 5′-TCCTAAACAGCGGTTGATGAGG-3′ 10 bp from VDE-DSB1; 5′-GCGAATGAAAGACGTCTTGG-3′ and 5′-CGGCCCTCTTAATTAGAACTTC-3′ 5.2 kb from VDE-DSB1; 5′ -CGCACATTTCCCCGAAAA-3′ and 5′-TGAAGACGAAAGGGCCTCG-3′.

Primers close to the *YCR047C* promoter regions were used to measure recovery of Spo11 DSB-associated sequences. The approximate distance from the PCR product to the nearest DSB is 0.6 kb (Buhler et al., unpublished data). A dilution series of genomic DNA from dAG206 strain was used to establish a standard curve. The ChIP enrichment was calculated as percentage of the target locus present in the immunoprecipitated sample relative to the amount in the starting input material. Corrected ChIP enrichment (Figure 6) was calculated based on estimates of the maximum proportion of probed chromatids likely to receive a DSB (P_max_). At *YCR047C* this value is 0.12 [68]. For VDE-DSB1 the correction value accounts for knowledge that up to 95% (Figure 2B) of all *arg4*-vde chromatids can receive a VDE-DSB1, and the proportion of chromatids with homology to the PCR primers and containing the *arg4*-vde allele varies with primer site. For primers close to and far from VDE-DSB1, the respective proportions of input chromatids containing the *arg4*-vde allele are 0.25 and 0.50 creating P_max_ values of 0.24 and 0.48. The corrected ChIP enrichment is the original ChIP enrichment value/P_max_ and thus reports on the ChIP enrichment as a proportion of chromatids that will receive a DSB during the time course.

To determine the ratio between corrected ChIP enrichment and proportion of chromatids expected to be single-stranded at the primer site, ssDNA values close to the Spo11-DSB at *YCR047C* were taken to be equal to the proportion of DSBs visible by Southern analyses in wild-type and *dmc1*Δ strains [68] (unpublished data). For the 10 bp from VDE-DSB1, the proportion of chromatids with ssDNA close to the break site was taken as the total proportion of *arg4*-vde chromatids present in resection bands at the relevant time points (Figure 3A; unpublished data). For the 5.2 kb from VDE-DSB1, the proportion of chromatids with ssDNA 8.5 kb from the break site was calculated (Figure 3A; unpublished data) and used as a conservative estimate of the proportion of chromatids that would be single-stranded where the primers lie. The DNA used to determine the proportion of chromatids with ssDNA was derived from different time courses used in ChIP experiments.

## Supporting Information

Table S1 List of the Diploid Yeast Strains Used in This Work (21 KB XLS)

### Accession Numbers

The accession numbers from the NCBI (http://www.ncbi.nlm.nih.gov) gene database for genes discussed in this paper are *DMC1* (856926), *HOP1* (854738), *MEK1* (854533), *NDT80* (−856524), *RAD54* (852713), *RFA1* (851266), *RED1* (850968), *SAE2* (852700), and *SPO11* (856364).

## Abbreviations

ChIP: chromatin immunoprecipitation
DSB: double-strand break
RNR: ribonucleotide reductase
RPA: replication protein A
SSA: single-strand annealing
ssDNA: single-strand DNA
VDE: *VMA1*-derived endonuclease
